# Exploring characteristics and common features of digital health in pediatric care in developing countries: a systematic review

**DOI:** 10.3389/fdgth.2025.1533788

**Published:** 2025-05-07

**Authors:** Anggi Septia Irawan, Bence Márton Döbrössy, Mengesha Srahbzu Biresaw, Arief Purnama Muharram, Szilárd Dávid Kovács, Edmond Girasek

**Affiliations:** ^1^ Institute of Behavioural Sciences, Faculty of Medicine, Semmelweis University, Budapest, Hungary; ^2^ School of Electrical Engineering and Informatics, Institut Teknologi Bandung, Bandung, Indonesia; ^3^ Metabolic Disorder, Cardiovascular and Aging Research Center, Indonesia Medical Education and Research Institute, Faculty of Medicine, Universitas Indonesia, Jakarta, Indonesia

**Keywords:** digital health, child health, low-resource setting, eHealth, mHealth interventions, health informatics

## Abstract

**Background:**

Digital health technologies have emerged as promising solutions to alleviate the scarcity of healthcare workers in developing countries. This systematic literature review aims to comprehensively explore the characteristics and common features of digital health interventions in pediatric care among parents and healthcare workers in these regions.

**Methods:**

A literature search was conducted on the PubMed and Scopus databases in January 2023, covering the period up to December 2022. The search adhered to the PRISMA guidelines. The PECOS format outlined by PROSPERO was used to determine the eligibility of systematic reviews and primary studies, with no restrictions on study designs or methodologies. Eligible articles comprised original research published in peer-reviewed open-access journals. The methodological quality of the included articles was independently assessed by authors using the CASP checklists to evaluate reporting quality.

**Result:**

The initial search yielded 1,334 publications, of which 16 met the inclusion and exclusion criteria for the review. These comprised 12 random control trials and 4 Mixed methods studies. The CASP criteria were applied to all studies, and those with a moderate to high level of methodological quality were included and reported. The reviewed publications described various types of Digital Health tools, with a majority (50%) of the studies conducted in Asia. The target users in the publications were diverse, with 37% focusing on mothers, 25% targeting pregnant women, and 19% targeting healthcare workers.

**Conclusions:**

The review highlighted a diverse range of tools, including mobile applications, websites, SMS, and phone calls, with a particular focus on breastfeeding, vaccination, and child growth. The findings emphasized the importance of healthcare worker participation, and the trust placed in information from relatives to influence the effectiveness of these tools. Moreover, the study underscored the need for intimate discussions when addressing sensitive topics like HIV. This review enhanced our understanding of the role of digital health tools in pediatric care in developing countries. It highlighted their potential to bridge healthcare gaps and promote wider access to quality care, addressing the challenges posed by limited healthcare resources in these regions.

**Systematic review registration:**

https://www.crd.york.ac.uk/prospero/display_record.php?ID=CRD42023383846, identifier: CRD42023383846.

## 1 Introduction

The global healthcare workforce is facing a critical shortage, with an estimated deficit of 17.4 million workers, a number first reported in 2013 (1). More recent assessments estimated a shortage of 15 million health workers in 2020, with a projected decline to 10 million by 2030 (2). Africa, for instance, had a mere 2.3 healthcare workers per 1,000 population, while the Americas boasted a significantly higher ratio of 24.8 per 1,000 (3). Astonishingly, only 1.3% of the world's health workers cater to the healthcare needs of those bearing 25% of the global disease burden. To foster inclusivity in healthcare, digital health technologies, encompassing the utilization of digital hardware and software in healthcare settings, present a promising solution (4).

The integration of digital health tools, including eHealth and mHealth, has shown great potential in enhancing healthcare delivery and increasing access to high-quality care (5). This review defines digital health as a specialized field that focuses on improving healthcare through the development and utilization of digital technologies. It elaborates on the notion of digital health by including individuals who make use of digital gadgets along with a broader array of intelligent devices and interconnected apparatus. Additionally, digital health incorporates diverse other implementations of digital technologies in the medical field, such as the Internet of Things, artificial intelligence, extensive data analysis, and robotics (6).

Digital health serves as a comprehensive framework that encompasses multiple components, such as electronic health (eHealth) and mobile health (mHealth). EHealth primarily revolves around computer-based applications, both online and offline, whereas mHealth specifically caters to applications specifically designed for mobile phones (7). These advanced technologies encompass a diverse range of applications, including mobile applications, websites, short message service (SMS), and phone calls, which enable the provision of various healthcare services such as information dissemination, teleconsultation, and digital health interventions. This is particularly relevant in developing countries, where limited access to clinical resources poses challenges for delivering optimal pediatric care (8).

Ensuring optimal pediatric care is paramount for nurturing children's growth and development, profoundly impacting their physical, social, and emotional well-being during their formative years (9). This holistic approach necessitates a concerted, team-based effort involving all healthcare professionals within the pediatric field (10), alongside attention to crucial factors such as adequate nutrition and environmental stimulation. However, meeting these standards can be challenging, particularly in resource-limited settings or areas with limited access to specialized care (9).

To overcome these challenges and enhance the delivery of optimal pediatric care, the integration of digital health technologies offers promising solutions. Digital health interventions, ranging from telemedicine for interprofessional consultations to telehealth initiatives for expanding physician capacity, present innovative opportunities to bridge geographical barriers and improve access to quality care. By embracing digital health strategies, healthcare providers can leverage technology to streamline workflows, enhance communication among multidisciplinary teams, and ultimately optimize outcomes for pediatric patients, regardless of geographical constraints or resource limitations (11).

Despite the growing recognition of the potential benefits associated with digital health in pediatric care, there remains a significant knowledge gap regarding the extent of utilization among parents and healthcare workers in developing countries. Additionally, limited understanding exists regarding the factors that influence the adoption and effectiveness of these tools in pediatric care settings. Addressing these research gaps is imperative for informing policy development, optimizing healthcare delivery, and ultimately enhancing the health outcomes of children residing in resource-constrained settings (12).

The World Health Organization has identified the utilization of information and communication technologies as one of the rapidly burgeoning domains in the current healthcare landscape. In March 2021, the United Nations Convention on the Rights of the Child noted the progressing integration of the digital realm into children's lives, underscoring the increasing significance of access to digital technology in shaping societal operations, health, and education. Pediatric care in developing countries faces numerous issues that require urgent attention to ensure the healthy development of the next generation. Problems such as stunting, malnutrition, and infectious diseases among infants are direct consequences of delayed healthcare worker interventions. In this context, digital health interventions emerge as a crucial solution (13).

This review aimed to explore the prevailing characteristics and common features linked to the implementation of digital health in developing countries, with a specific focus on pediatric care. Within this framework, developing countries are defined as middle-to-low-income nations, as per the World Bank classification utilizing gross national income per capita (14). The research questions aimed to discern the outcomes of digital technology interventions in these resource-constrained settings. The discoveries from this review will not only shed light on these crucial aspects but also contribute valuable insights for decision-makers, medical practitioners, and researchers. These insights are pivotal for those seeking to encourage the successful integration of eHealth and mHealth tools into pediatric care in developing nations. The collective findings will aid in informed decision-making and the development of effective strategies to optimize the utilization of digital health technologies in enhancing healthcare outcomes for children in resource limited settings.

## 2 Methods

### 2.1 Literature search

A literature search was conducted on the PubMed and Scopus databases in January 2023, covering a period up to December 2022. The search followed the PRISMA (Preferred Reporting Items for Systematic Reviews and Meta-Analyses) guidelines (15). A predetermined protocol was registered on the International Prospective Register of Systematic Reviews (PROSPERO) with the registration number CRD42023383846. The protocol outlined the methods for data searching, inclusion criteria, and data extraction. More information about the registration can be accessed at: https://www.crd.york.ac.uk/prospero/display_record.php?ID=CRD42023383846.

### 2.2 Research questions

This review aimed to identify the key characteristics and common features of digital health interventions in pediatric care within developing countries, defined as middle to low-income nations according to the World Bank classification. The primary research questions addressed in this study were: (1) what types and characteristics of digital health interventions are commonly implemented in pediatric care within resource-constrained settings? (2) what are the primary outcomes associated with these interventions in terms of child health and healthcare delivery. To search for relevant literature, a set of keywords was generated based on research questions. The keywords included terms related to child growth, pediatric health, and various digital technologies. Additionally, terms related to developing countries and different types of mobile devices were included in the search. We used the following grouped search terms: Child AND Growth AND Monitor AND Health AND Pediatric AND (short message service OR smartphone OR cellular telephone OR mobile phone OR personal digital assistant OR mobile app OR mobile application OR Digital Health OR eHealth OR mHealth) AND (developing country OR low-income country OR middle-income country OR Low and Middle Income Countries OR Low Income Countries OR Middle Income Countries OR LMIC OR LIC OR MIC).

### 2.3 Study selection

To determine the eligibility of systematic reviews and primary studies, the PECOS format outlined by PROSPERO was followed. All eligible research reports published and available in January 2023 were included based on the following criteria:

The inclusion criteria for this overview required that studies meet the following criteria's:

• Participants: This study encompassed individuals falling into several categories: children, parents with children under 5 years old, pregnant women, and healthcare workers located in developing countries, third-world countries, or low-to middle-income countries (as per OECD classification). These groups were selected as key stakeholders in pediatric healthcare delivery within resource-constrained settings. Children, especially those under the age of 5, signify a critical developmental stage where digital health interventions can profoundly impact healthcare outcomes. Additionally, the inclusion of pregnant women was deemed essential due to the interconnectedness of prenatal care with pediatric care, as it directly influences the health and well-being of both the mother and the child
• Exposure: Studies that utilized mobile phone applications, Short Message Service (SMS) messages, or web-based interventions for pediatric care.
• Comparator/Control: Studies with a community that did not use mHealth and/or eHealth interventions.
• Outcomes: Studies report on various primary outcomes related to the impact of mHealth/eHealth on pregnancy, parenting, child growth measurement, and support for healthcare workers.

No restrictions were placed on specific study designs or research methodologies. The eligible articles included original research published in peer-reviewed open-access journals. Two independent investigators (SDK and ARM) assessed the article's eligibility. Any discrepancies were resolved through discussion, involving a third reviewer (MSB) to reach a consensus.

### 2.4 Screening process

The initial screening of publications retrieved from the PubMed search using the specified keywords focused on evaluating the relevance of titles and abstracts. Publications were considered potentially relevant if their title and abstract were connected to the topic of the review. In the second screening phase, the full text of the publications deemed potentially relevant in the first screening was obtained and thoroughly assessed. Publications that met the inclusion criteria mentioned earlier were classified as relevant and selected for review. Following the screening process, the publications were analyzed using content analysis, which involved identifying categories and synthesizing the classified information within each category.

### 2.5 Data extraction and analysis

Data extraction and analysis were performed by four reviewers (ASI, SDK, APM, MSB). The following categories were extracted: sample size, setting, authors, publication year, countries, number of participants, location of the study, app name and platform, purpose of the app, intervention period, study limitations, and outcomes. The articles were categorized based on the App's functionality, such as providing information, providing feedback, monitoring behavior change, or alarming the user regarding health behavior. Disagreements were resolved through consensus-based discussions. Given the heterogeneity of the systematic reviews and primary studies, including differences in study designs, participants, settings, Digital health platforms used, measurement tools, and outcomes of interest, it can be concluded that conducting a meta-analysis of the results was not feasible.

### 2.6 Study quality assessment

Four authors (ASI, SDK, APM, and MSB) independently assessed the methodological quality of the included articles using the Critical Appraisal Skills Program (CASP) to evaluate reporting quality (see Supplementary Material S2). In cases of missing or unclear data, the authors of the papers were contacted to request additional information. If any disagreements arose between the two reviewers, a fifth reviewer was involved to reach a resolution. The results of the critical appraisal were reported narratively and in tabular form.

## 3 Result

**Figure 1:**
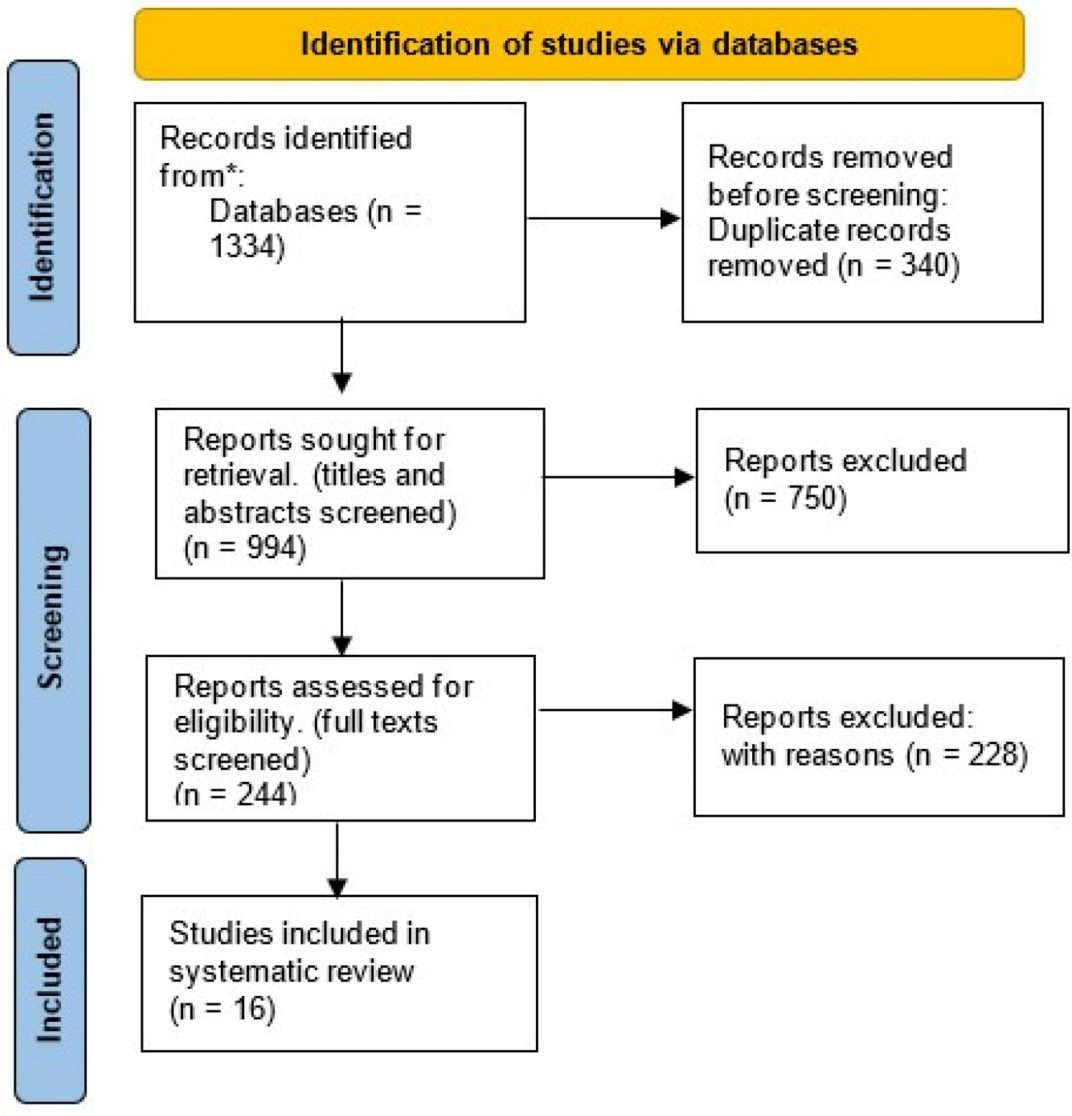
Flow diagram for systematic selection of research articles from databases.

A flow diagram illustrating the study selection process is presented in Figure 1. The initial database searches yielded 1,334 articles, which were then screened for duplicates, leading to the exclusion of 340 duplicate entries. Following this, 750 articles were excluded based on their titles and abstracts, as they did not meet the three main inclusion criteria: (1) focus on developing countries, (2) digital health interventions, and (3) pediatric care apps. After the second round of screening, the full texts of the remaining publications were reviewed in detail. From this, 16 studies were identified as meeting all inclusion criteria and were selected for review. These studies focused on mHealth or eHealth interventions within pediatric care, including maternity, postnatal, and child development perspectives. The study participants in these studies were categorized into four groups: children, parents, pregnant women, and healthcare workers (Figure 1).

### 3.1 Result of quality assessment

All 16 studies consist of 12 random control trials and 4 mixed methods studies/qualitative-quantitative studies (see Supplementary Material S1). We used two CASP checklists appropriate to the studies. All studies with a moderate to high level of methodological quality were included and reported in this study. Regarding the mixed methods studies using the one-checklist approach, all of them fulfilled the CASP criteria, except for one checklist question about the researcher-participant relationship. All mixed methods’ studies did not clearly mention the researcher-participant relationship.

From randomized controlled trials (RCT) research, one concern that was not mentioned in all the studies was the absence of blind research in the intervention process. Three studies did not provide clear information on whether participants were unaware of the specific interventions they received, and four studies did not address this aspect at all. Furthermore, four studies did not indicate whether the researchers delivering the interventions were also blinded to them. According to the CASP checklist, all articles met adequate and satisfactory standards, except for a minor study that did not fully satisfy all the criteria. Independent reviewers agreed that all the included studies demonstrated a satisfactory level of methodological quality. This suggests that the findings can be reasonably generalized and applied to the study population, with a strong level of agreement among independent evaluators.

### 3.2 General study characteristics

**Figure 2:**
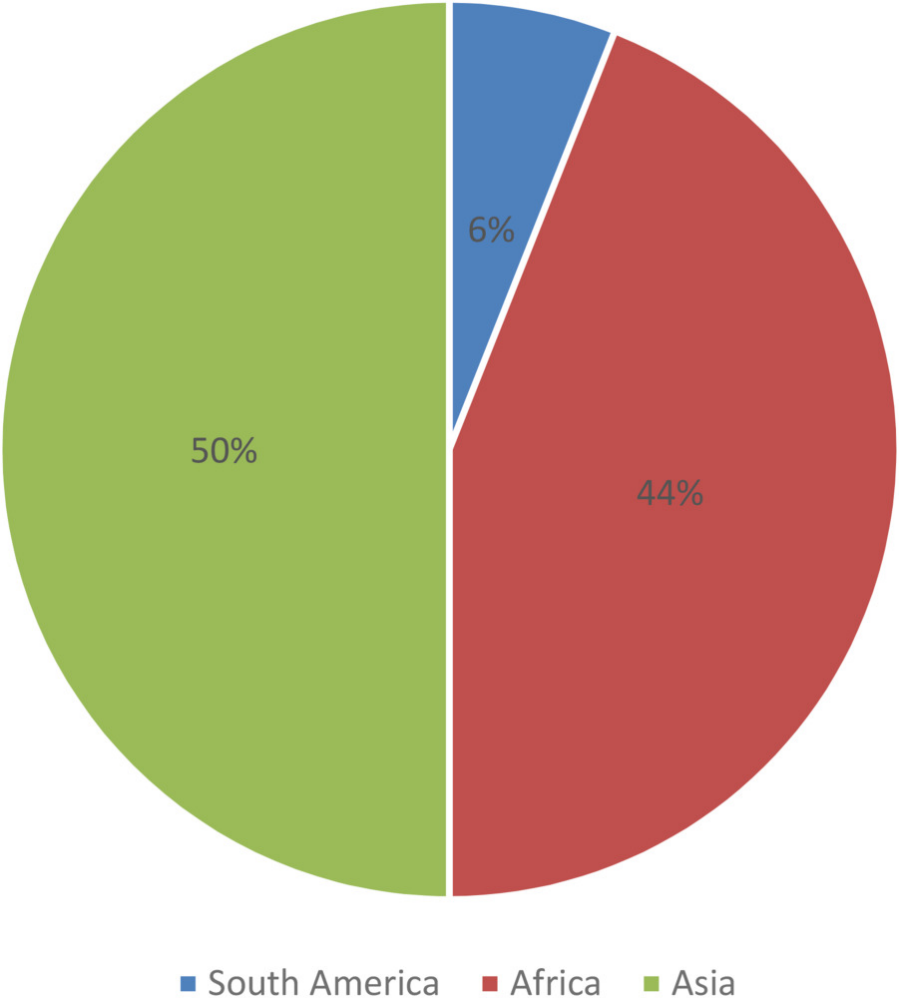
Studies included by continent (*N* = 16).

The 16 selected studies were published in peer-reviewed journals within the past decade (see Supplementary Material S2). Figure 2 shows that all of these studies were conducted in developing countries, with eight studies taking place in various Asian countries such as China (16, 17), Thailand (18), Vietnam (19), Indonesia (20), and India (21, 22). Moreover, seven studies were conducted in different African countries including Ethiopia (23, 24), Kenya (25, 26), and Malawi (27), while one study was carried out in Guatemala, Central America (28).

Most of the studies (13 out of 16) focused on the fields of medicine and public health, while the remaining three explored topics related to nursing and biotechnology. In terms of sample sizes, four studies had sample sizes exceeding 1000 participants, three studies had sample sizes ranging from 500 to 1,000, six studies (16.6%) included sample sizes between 100 and 500, and three studies had sample sizes below 100. All the studies were conducted in community settings, as indicated in Table 1. The research participants predominantly consisted of parents (7 studies) and pregnant women (4 studies), followed by healthcare workers (2 studies), children (1 study), and a combination of healthcare workers and parents (2 studies).

**Table 1:** General study characteristics (*N* = 16).

Variables	Categories	*N* (%)
Type of Studies	Published journal	16
Major field of researchers	Public health	6
	Nursing	1
	Medicine	8
	Biotechnology	1
Sample size	Under 100	3
	100–500	6
	500–1,000	3
	>1,000	4
Research Participants	Parents	7
	Healthcare worker	2
	Children	1
	Pregnant women	4
	Healthcare workers and parents	2
Setting	Community	16

### 3.3 Characteristic of mHealth and eHealth for pediatric care

Table 2 provided an overview of platform types, target users, and main features in digital health technology across various countries. The first category highlighted the types of tools employed, with mobile applications and SMS being the most widely utilized platforms. Out of the 16 studies reviewed, 14 (88%) employed either SMS or mobile applications. SMS commonly served as a reminder tool, while mobile apps were used for tele-consultation and delivering health information.

**Table 2:** The classification of platform types, target users, and main features in digital health technology across various countries.

Classification category	Subcategories	*N* (%)	Countries	First author, year published (see Supplementary Material S1)
Type of platform	Mobile application	7 (44%)	China, Thailand, Vietnam, Indonesia, India, Malawi	Duan, 2020
				Nurhaeni, 2021
				Chirambo, 2021
				Areemit, 2020
				Chen, 2016
				Wu, 2020
				Wu, 2021
				Modi, 2019
	SMS/Text message	7 (44%)	Ethiopia, Kenya, Guatemala	Mekonnen, 2021
				Kebaya, 2014
				Unger, 2018
				Kinuthia, 2021
				Domek, 2019
				Wakadha, 2013
				Mekonnen, 2021
	Phone call	1 (6%)	India, Kenya	Kebaya, 2014
				Patel, 2018
	Combine mobile application and Website	1 (6%)	India	Modi, 2019
	Combine phone call and SMS	1 (6%)	India	Patel, 2018
Target users	Mother	6 (37%)	Ethiopia, Indonesia, China, Kenya,	Mekonnen, 2021
				Nurhaeni, 2021
				Chen, 2016
				Unger, 2018
				Kinuthia, 2021
				Wakadha, 2013
	healthcare worker	3 (19%)	Kenya, Thailand, China	Kebaya, 2014
				Areemit, 2020
				Chen, 2016
	Pregnant women	4 (25%)	China, Malawi, India	Wu, 2021
				Chirambo, 2021
				Patel, 2018
				Modi, 2019
	Children	1 (6%)	Guatemala	Domek, 2019
	Parent	2 (12%)	Thailand, Vietnam	Areemit, 2020
				Duan, 2020
	Combine parent and healthcare provider	1 (6%)	Ethiopia	Mekonnen, 2021
The main feature	Vaccination Reminder	8 (50%)	China, Thailand, Indonesia, Ethiopia, Kenya, Guatemala	Mekonnen, 2021
				Nurhaeni, 2021
				Kebaya, 2014
				Domek, 2019
				Areemit, 2020
				Wakadha, 2013
				Chen, 2016
				Mekonnen, 2021
	HIV prevention	2 (12%)	Kenya	Kebaya, 2014
				Kinuthia, 2021
	Growth monitoring	2 (12%)	China, Thailand	Kebaya, 2014
				Areemit, 2020
				Wu, 2021
	Child nutrition	2 (12%)	China	Kebaya, 2014
				Wu, 2021
				Chen, 2016
	Pediatric surveillance system	1 (6%)	Malawi	Chirambo, 2021
	Breastfeeding	4 (25%)	Vietnam, India, Kenya	Chen, 2016
				Unger, 2018
				Patel, 2018
				Duan, 2020
	Maternal, neonatal, and child health (MNCH) services	1 (6%)	India	Modi, 2019

We provided an analysis of the target users of the interventions described in these studies. From the 16 studies, different publications focused on varying target populations: 6 studies (38%) targeted mothers, 4 studies (25%) targeted pregnant women, and 3 studies (19%) focused on healthcare workers. In addition, one publication (6%) focused on children, one (6%) on parents, and one (6%) on both parents and healthcare workers. The specific purposes of these interventions varied, with 8 studies (50%) focusing on vaccination, 4 (25%) on breastfeeding, and the remainder addressing issues such as HIV prevention, growth monitoring, and child nutrition (Table 2).

These diverse approaches exemplified the range of digital health used in childcare across different countries, by encompassing computer-adapted mobile application programs, phone calls, and SMS reminders. From Table 2, another widely implemented program involved SMS reminders, particularly prevalent in African countries (23, 25, 26, 29, 30) and Guatemala (28). For instance, a vaccination study in Kenya employed a reward system with monetary incentives (25). These systems, based on the use of eHealth/mHealth tools, effectively drive tangible changes in health-related behaviors. Participants can track their progress under the guidance of healthcare workers, which serves as a motivational factor.

Furthermore, for specific purposes like private and personalized consultations, two studies indicated the utilization of tele-consultation telephone calls for preventing HIV transmission in infants and addressing breastfeeding concerns (29, 30). These tele-consultations offer an effective solution for meeting specific healthcare needs in remote or inaccessible areas. Overall, the diverse utilization of eHealth and mHealth tools, including computer-adapted mobile applications, phone calls, SMS reminders, and tele-consultation, showcases the multifaceted strategies employed in childcare.

### 3.4 Outcomes of pediatric care studies

In this section, we presented a comprehensive overview of the outcomes and limitations of all included studies (see Supplementary Material S3). Each study type and its associated eHealth/mHealth tool provide specific insights into their effectiveness.

#### 3.4.1 Vaccination reminder

In the cross-sectional quantitative study conducted in Ethiopia, the use of SMS/text message reminders for vaccination showed positive results, with 75% of participants expressing the desire to receive reminders before the vaccination due date. However, the study acknowledged limitations in generalizability, as it was conducted on individuals with mobile phones and vaccine appointments, potentially excluding those in rural areas. The study also highlighted the risk of marginalizing populations without access to mobile phones (24).

The quantitative cross-sectional research in Indonesia focused on the use of a mobile application for vaccination. It found that mothers with a supportive attitude toward vaccination and those who used the mobile app were more likely to complete basic vaccination for their children. However, the study had limitations in terms of respondent selection and did not explore specific features and user perspectives of the application (28).

A similar study conducted in Guatemala utilized SMS vaccination reminders for patient visits. The results indicated comparable high rates of visit completion in both the intervention and usual care groups. However, participants in the intervention group tended to present earlier for their scheduled visits. In addition, there was a high level of parental satisfaction reported with the SMS reminders. Despite these positive outcomes, it's worth noting that this randomized controlled trial did not observe an improvement in overall visit completion rates with the intervention (28).

#### 3.4.2 Child growth monitoring

Mixed-method study investigated the use of mobile applications in Thailand for monitoring child growth, facilitating communication between parents and physicians. However, it is important to note that certain data, such as physician or parent visit records, were not captured in this study, indicating a limitation in our data collection process (18).

In Kenya, a randomized control trial examined the effectiveness of phone calls as an intervention. The study found higher adherence to infant NVP (Nevirapine prophylaxis) and increased retention in care among participants in the intervention arm. However, limitations such as self-reporting bias, confidentiality risks, and the impact of a health care provider strike affected the study's outcomes (29).

#### 3.4.3 Breastfeeding and nutrition support

A mixed-methods study conducted in rural China evaluated the use of a mobile application for breastfeeding and complementary feeding. The research revealed limited knowledge among pregnant women and caregivers in the research setting, indicating a need for accurate information sources on infant feeding and child nutrition. The study also highlighted the widespread use of smartphones and the WeChat app among pregnant women and mothers in the area (31).

#### 3.4.4 Maternity and infant health

Two randomized controlled trials (RCTs) conducted in India examined the effectiveness of using SMS and a combination of mobile applications and websites for improving lactation counseling via cell phones. The findings demonstrated that these interventions were highly beneficial, providing frequent and sustained support to pregnant and lactating mothers. Besides, the ImTeCHO mobile and web-based application were found to enhance the coverage and quality of maternal, newborn, and child health (MNCH) services. However, it's important to note that one of the studies was unblinded, potentially introducing bias into the results. Furthermore, another reported inaccuracy in the mothers' reporting of events during pregnancy, and the intervention's duration was limited to 12 months (21, 22).

#### 3.4.5 Pediatric surveillance system

In a cluster randomized trial in Malawi, the use of a mobile application for community case management (e-CCM) showed potential benefits in improving decision-making at the health surveillance assistant (has) level. The study highlighted the importance of investing in a comprehensive surveillance system as a crucial step in reducing child mortality rates and improving overall child health outcomes. This study faced challenges in evaluating the independent impact of e-CCM because health surveillance assistants (HSAs) continued using paper-based tools alongside the mobile application.

## 4 Discussion

This comprehensive systematic literature review offers valuable insights into the utilization of eHealth and mHealth tools in pediatric care, as well as the factors influencing their usage. Out of the studies assessed using the CASP (Critical Appraisal Skills Program) criteria, 16 met the necessary inclusion criteria. However, one aspect highlighted in the CASP assessment that deserves further consideration is the importance of addressing blind research. Additionally, establishing a clear comprehension of the connection between participants and researchers is crucial to minimizing the risk of bias in research (15).

In developing countries, various types of eHealth and mHealth tools are utilized for pediatric care by both parents and healthcare workers. These tools include mobile applications, websites, SMS, and phone calls. Examples of such tools include computer-tailored pediatric care, teleconsultation, digital health information, and telehealth programs. While most publications focus on the broad applications of eHealth and mHealth tools for parents, only a few studies provided support for healthcare workers in their pediatric practice (32).

Karamagi et al.'s research stands out by uncovering 738 digital health interventions across Sub-Saharan Africa. Their findings revealed concentrations in certain countries, considerable redundancy, and a troubling emphasis on data mining rather than actual service delivery. The findings underscored the urgent need to re-strategize digital health development, align investments with health system strengthening principles, and encourage collaboration for sustainable implementation. Furthermore, the study emphasized the challenges of uncoordinated large-scale implementation, highlighting the importance of aligning digital health investments with national strategies and promoting coordination, sustainability, and knowledge sharing in digital health interventions across the region (32).

Pediatric care Apps in developing countries often emphasize features related to breastfeeding, vaccination, and child growth. However, there are differences observed in African countries where additional features are developed for HIV prevention in maternity and mother with infant. In these cases, private and personal interactions through phone calls and SMS are commonly utilized, along with conventional mobile phone usage (33). This is specific to African countries compared to other developing regions such as Asia and Central America, reflecting challenges related to internet infrastructure, competencies, and digital literacy that hinder the wider adoption of mobile phones (34). Besides, in Kenya, incentives were applied to increase vaccination participation rates, as mentioned in a WHO report (25).

In China, WeChat has firmly established itself as the leading social application with a staggering user base of over 1.1 billion active users every month. Currently, this application has become a central tool utilized in pediatric care settings. Among the three studies mentioned, all of them employed WeChat with certain modifications based on their specific objectives. However, the findings indicated that relying solely on applications was insufficient to fully support pediatric care, as Chinese individuals still place strong trust in information and references provided by their relatives, particularly in the context of breastfeeding and vaccination. Furthermore, mobile applications can be effective if healthcare workers actively participate in the program (35).

In other Asian countries, computer-tailored applications were commonly used, while India utilized a combination of three platforms: mobile applications, SMS, and web-based platforms. Generally, the focus in Asia is like other developing countries, lying on vaccination, breastfeeding, and growth development (21, 22).

Overall, the analysis of these studies suggests that digital health tools have the potential to positively impact the child healthcare outcomes. However, there are various limitations and challenges that need to be addressed, such as generalizability, technological barriers, data completeness, and patient record management. Despite these limitations, the studies demonstrated promising results in terms of vaccination coverage, immunization completion, breastfeeding adherence, and improved decision-making at the community level. Further research and tailored strategies are necessary to maximize the effectiveness and implementation of these tools in child healthcare.

## 5 Limitations

The studies assessed in this review have limitations that hinder their generalizability in the countries they represent. These limitations encompass variations in sampling methods, total participant numbers, the high diversity of populations, and country-specific infrastructures. Consequently, caution should be exercised in interpreting the results of this study, as they may not fully capture the landscape of pediatric care in developing countries. Furthermore, the evaluation of technology was confined to published studies, with individual applications or programs not being assessed. Moreover, the use of English-language queries may have resulted in articles only available in English, potentially limiting the generalizability of our findings. It is recommended that future studies undertake a systematic review of published articles and include a comprehensive assessment of digital technology programs. This is necessary to achieve a more holistic understanding of digital technology development and its implications for pediatric care in developing countries.

## 6 Conclusion

In conclusion, this systematic literature review provided valuable insights into the characteristics of digital health in pediatric care in developing countries. The review highlighted the diverse range of tools used, including mobile applications, websites, SMS, and phone calls, with a focus on breastfeeding, vaccination, and child growth. Regional variations were observed, such as the central use of WeChat in China and the adoption of computer-tailored applications in other Asian countries.

The findings underscored the importance of healthcare worker participation, and the trust placed in information from relatives in influencing the effectiveness of these tools. The study also emphasized the need for intimate discussions when addressing sensitive topics like HIV in maternity.

Overall, this review contributed to a better understanding of the role of eHealth and mHealth tools in pediatric care in developing countries. It highlighted the potential for these technologies to bridge healthcare gaps and to promote wider access to quality care.

However, further research is needed to comprehensively assess digital technology programs (the applications program) and their impact on pediatric care outcomes. By addressing these considerations, we can effectively harness the benefits of eHealth and mHealth tools to enhance pediatric care in resource-constrained settings.

## Data Availability

The raw data supporting the conclusions of this article will be made available by the authors, without undue reservation.
