# Supplementary material for: Exploring characteristics and common features of digital health in pediatric care in developing countries: a systematic review
Source: Front Digit Health. 2025 May 7;7:1533788. doi: 10.3389/fdgth.2025.1533788 (PMC12092402; doi:10.3389/fdgth.2025.1533788)
Supplement: Supplementary file 3 [file Table3.docx]

**Supplementary material 3**

Limitations and outcomes of the study

| Country | Study type | E-Health/M-Health | Study limitation | Study outcome | Reference index (supplementary file 1) |
| --- | --- | --- | --- | --- | --- |
| Ethiopia | A cross-sectional quantitative study design | SMS/Text message | The findings may not apply to the whole country, especially rural areas, as the study only included people with mobile phones and vaccine appointments. Using mobile phone reminders may exclude those without phones, which is a potential drawback. The study also didn't address other barriers to implementation or explore users' intentions further, so more research is needed. | Around 75% (270) preferred vaccination reminders a day before the scheduled date. Factors predicting text message usage for vaccination included being mothers aged 35 years or older (AOR=0.35; 95% CI: 0.15 to 0.83), higher education (AOR=4.43; 95% CI: 2.05 to 9.58), longer mobile phone use (AOR=3.63; 95% CI: 1.66 to 7.94), perceived usefulness (AOR=6.37; 95% CI: 3.13 to 12.98), and perceived ease of use (AOR=3.85; 95% CI: 2.06 to 7.18). | 1 |
| Indonesia | Quantitative cross-sectional research | Mobile Application | The study had limitations in finding respondents using PrimaKu. Additionally, it did not examine specific immunization features or assess users' perspectives on the advantages and disadvantages of the application. | Around 44.5% of children had completed basic immunization. Mothers with a supportive attitude towards immunization were 3.58 times (95% CI 1.49-8.57, p<0.003) more likely to ensure complete immunization. Additionally, mothers using the mobile app had a 3.23 times higher likelihood (95% CI 1.18-8.87, p<0.034) of achieving full immunization for their child. | 2 |
| Kenya | Randomized controlled trials (RCT) | Phone Call | Self-reporting adherence to infant nevirapine and exclusive breastfeeding may not provide accurate information. Future research should consider incorporating facility-level data to verify nevirapine adherence and exclusive breastfeeding. Additionally, a limitation of the study was the shared use of mobile phones in some households, which posed a risk to confidentiality. | At the 6-week follow-up, 90.7% (68) of participants in the intervention group adhered to infant NVP prophylaxis, compared to 72% (54) in the control group (p = 0.005). The intervention group also had significantly higher retention in care. At 6 weeks, 78.7% (59) of mother-infant pairs in the intervention arm attended scheduled visits on the appointment date, while in the control arm, only 58.7% (44) did so (p = 0.009). By 10 weeks, the revisit rates were 69.3% (52) in the intervention group and 37.3% (28) in the control group among the evaluated 150 mother-infant pairs (p < 0.001). | 3 |
| China | Mixed method (qualitative-  quantitative) | Mobile Application | Study takes place in one rural county in Lack of qualitative interview among mothers with children aged 6-23 months | Both pregnant women and caregivers had limited knowledge about exclusive breastfeeding and complementary feeding in our research setting in rural China; The key IYCF indicators were suboptimal and did not improve over the years. Absence of accurate information sources on infant feeding and child nutrition; Caregivers mostly received feeding information from their relatives and friends. | 4 |
| Malawi | Cluster random trial | Mobile application | HSAs continued to use the paper tool during the intervention phase, which meant that we could not assess the independent impact of e-CCM but rather its benefit; Lack of unique patient identifiers and incomplete records at health care facilities. | Although the effects on decreased hospital admission and decreased repeat consultation were suggestive but inconclusive. They support the hypothesis that the e-CCM tool improved decision-making at the HSA level. | 5 |
| China | Randomized controlled trials (RCT) | Mobile Application | As the Chinese Lunar Year and the COVID-19 outbreak occurred in January 2020 in China, interviewers could not conduct follow-up data collection during that time. | Antenatal plus postnatal WeChat breastfeeding education was associated with higher rates of exclusive and predominant breastfeeding in the early postnatal period. | 6 |
| Kenya | Quantitative | SMS/Text message | Effects of SMS alone could not be determined; Free maternity care was rolled out in early 2013 and resulted in 98% facility delivery in all randomized clinical trial arms. | Both SMS intervention arms in our study resulted in higher rates of exclusive breastfeeding at 10 and 16 weeks. Women in the 2-way SMS arm were significantly more likely to adhere to exclusive breastfeeding recommendations for up to 24 weeks. Both 1-way and 2-way SMS approaches increased early uptake of contraception by 16 weeks postpartum, but the difference was not statistically significant. | 7 |
| Kenya | Randomized controlled trials (RCT) | SMS/Text message | Abstraction of clinic and VL data was challenging due to completeness and data quality issues. Changes in VL testing limits and clinic records (transitioning from paper to electronic and occasional lapses back to paper) occurred during the study. Nurses faced difficulties entering appointment dates for visit reminder SMS due to unavailable clinic records. | During pregnancy to 2 years postpartum in PMTCT programs, 1-way and 2-way SMS did not significantly change maternal clinic engagement, retention, viral non suppression, or infant HIV-free survival; 2-way SMS was associated with significantly increased viral suppression. | 8 |
| Guatemala | Randomized controlled trials (RCT) | SMS/Text message | Improvement in overall visit completion rates with the intervention is not seen, partly due to selection bias in enrolling children as they presented for their first immunization visit; Errors with automated SMS system. | Our study results showed similarly high rates of visit completion for both the intervention and usual care groups, but intervention participants presented earlier for their scheduled visits and expressed high parental satisfaction with the SMS reminders. | 9 |
| Thailand | Mixed method (qualitative-  quantitative) | Mobile application | The intervention did not lead to an overall improvement in visit completion rates, possibly due to selection bias during enrollment at the first immunization visit. Errors in the automated SMS system resulted in some participants not receiving all messages, and there were instances of incorrect visit dates or telephone numbers entered by patients or staff. Visits to non-study clinics were not recorded in this study. | This is the first study to describe the development and evaluation of a mobile app for child health supervision by comparing the congruence of parent-physician growth assessments and parental evaluation in Thailand, KhunLook, a Thai mobile app for child health supervision, was developed, validated for growth assessments, and was found to be well accepted for ease-of-use by parents. | 10 |
| Kenya | Quantitative | SMS/Text message | Studies area was limited | Sms reminder and cash transfer support potentially improve vaccination rate. | 11 |
| China | Randomized controlled trials (RCT) | Mobile application | The limited impact of EPI app may have been due to implementation of text messages in both the intervention and control group. The vaccination coverage results may be biased due to vaccination coverage in the study area was already relatively high. The study cannot be generalized for all health workers with a range of technology capabilities. | We found that using the EPI app and text messaging reminders increased child vaccination coverage. However, EPI alone may not improve child vaccination coverage effective. Improved work efficiency of village doctors was also an important impact of the app. | 12 |
| India | Randomized controlled trials (RCT) | SMS/Text message | Unblinded study.  Another limiting factor was that the intervention was not designed to assess the effectiveness of different frequencies of contact with the women, on exclusive breastfeeding. | This study found that lactation counselling using cell phones proved to be a very useful tool for frequent and sustained support to pregnant and lactating mothers. | 13 |
| India | Randomized controlled trials (RCT) | Combine mobile application and website | The limitations of the study include potential risk of inaccuracies in reporting events that occurred during pregnancy by the mothers and the duration of intervention being 12 months, which might be considered short. | The ImTeCHO-mobile-and web-based application improved coverage and quality of MNCH services in hard-to-reach areas. | 14 |
| Ethiopia | Qualitative | SMS/Text message | This study was conducted in the urban community which may limit the transferability of the findings to different contexts. Self-reported data is subject to social desirability bias, and thus participants might have exaggerated responses. We also acknowledge that data saturation is a disputable concept and novel themes may have emerged from further interviews. | Mobile phone text message reminders for child vaccination service are acceptable by clients. Mothers who received the mobile text message reminders found it clear and understandable. ICT Infrastructure (phone ownership, network, and electricity), literacy, and language-related issues were considerable barriers to implementation. In this study, security and confidentiality concerns were not a barrier to the implementation of SMS-based mHealth initiatives for the EPI program in the study setting. Stakeholder’s collaboration, willingness of clients to pay for the mobile text message reminders, and provision of orientation/training to end-users before actual implementation are the facilitators for implementation. | 15 |
| Vietnam | Randomized controlled trials (RCT) | Mobile Application | The intervention and control group may differ in terms of socio-demographic characteristics, potentially limiting comparison between the two groups on breastfeeding outcomes. Nonetheless, a statistical analysis plan will be developed, including adjustment for baseline covariates. | The app will be developed to promote early initiation of breastfeeding and exclusive breastfeeding for the first six months of life. In particular, the app will seek to overcome the low rate of breastfeeding following caesarean section. | 16 |
